# Health expenditure convergence and the roles of trade and governance in Africa

**DOI:** 10.1186/s13690-023-01078-3

**Published:** 2023-04-28

**Authors:** Ariane Ephemia Ndzignat Mouteyica, Nicholas Ngepah

**Affiliations:** School of Economics, University of Johannesburg, Johannesburg, South Africa

**Keywords:** Health expenditures, Convergence, Trade, Governance quality, Africa, Convergence club

## Abstract

This study investigates the convergence hypothesis in health expenditures in 40 African countries over the 2000–2019 period. The new non-linear dynamic factor model is used on panel data extracted from the World Development Indicator and the World Governance Indicator. We consider two categories of health expenditures: the domestic general government health expenditure as a percentage of government expenditure and the domestic general government health expenditure per capita. The results show the absence of full panel convergence for the two indicators used. However, there is evidence of convergence clubs. The overall marginal effect of the control variables is consistent with the existing literature. The results further show a strong influence of trade in attaining convergence among the clubs for both models. However, governance quality only affects the probability of converging in a club for the general government health expenditure as a percentage of government expenditure model. The findings suggest that policies on promoting health expenditure convergence should pursue initiatives that encourage trade. Efforts should also be targeted to improve the quality of governance.

## Introduction

Healthcare systems across Africa are weak and underfunded. Although the continent made up 11 percent of the world’s population and 24 percent of the global disease burden, it only counted for just one percent of the global health finance [1]. Furthermore, The UN Economic Commission for Africa pointed out that the continent has an annual public health financing shortfall estimated at $66bn (Knight Frank Research, 2020). Many African countries face challenges in raising public funds for health, mainly because of the mechanisms and strategies used to finance the health sectors. For instance, about 40 percent or more of total health expenditure in most countries originate from out-of-pocket (OOP) payments. Moreover, government health expenditure per capita is below US$25 in several countries [2].

Low investment in the health sector is of great concern in Africa, particularly considering that the continent bears the double burden of communicable and non-communicable diseases. In addition, Africa has the highest mortality and morbidity rates in the world due mainly to HIV/AIDS. Even when external resources on health are critical, governments need to allocate more funds to their health sectors. In this regard, African leaders have adopted pro-health commitments to increase government spending on health and achieve better health outcomes. For instance, in 2001, most countries agreed to allocate at least 15 percent of the government budget to the health sector. The 2001 Abuja Declaration constitutes the landmark, which was later reaffirmed in the 2003 Maputo Declaration. However, only a few countries, such as Rwanda, Botswana, and Zambia, met the Abuja target in 2010. During the same year, over 12 countries reduced their relative government allocations to health, while in others, the trend remained unchanged [2].

The levels of government health spending in African countries largely depend on political decisions regarding the priorities of government actions and interventions. Countries with a high political will in health financing reforms and interventions have made significant progress in mobilizing resources for their health sectors. However, despite several efforts made by these countries, the question of efficiency and transparency in the use of resources remains a major concern in Africa. In addition, bad governance and weak accountability mechanisms have mainly undermined the effective implementation of health policies, including the lack of control of beneficiaries over the use of public funds and inefficient control of the governments over behaviors of service providers such as corruption [3].

Furthermore, the capacity of countries to generate more public funds is a question that lies outside the health sector. In this light, [4] showed that trade openness boosts economic growth, leading to additional fiscal space for the health sector. Additionally, [5] revealed that trade openness improves international integration and brings financial benefits such as grants and aid to the health sector. However, the relationship between trade openness and health financing has received limited attention in the empirical literature, particularly in Africa. Similarly, little is known about the link between governance and health spending.

Moreover, although the disparities in health spending between African countries are well known, little is known about whether the differences have decreased over time. Existing studies on the current health expenditure data have shown several limitations in understanding the accurate trajectories of health expenditure disparities between African countries that have experienced substantial health financing reforms over the past two decades [2]. The existing literature on health expenditure convergence mainly focuses on developed countries. To the best of our knowledge, only two studies in the field have concentrated on SSA, namely [6] and [7], while the other two studies, [8] and [9] have investigated health expenditure and health outcome convergence in SADC and ECOWAS, respectively. But these studies used standard convergence measures that have several flaws as discussed in the literature review.

The present study, therefore, examines the convergence in health expenditures in 40 African countries from 2000 to 2019. We use the new methodology developed by [10, 11]. This method is based on a non-linear time-varying factor model that allows the identification of possible convergence clubs. It also incorporates the possibility of transitional divergence among countries. The analysis of health expenditure convergence in Africa has several policy implications. Firstly, it may lead to the identification of potential financial risks caused by a global pandemic, such as the Covid-19 crisis. Secondly, it allows the assessment of the outcomes emerging from common health policies and strategies implemented at the continental, regional, and country levels, which is essential for policy and planning concerning the health sector.

Furthermore, assessing the progress in health expenditure is vital for developing and implementing future health policies as it may suggest ways to achieve the Sustainable Development Goals targets within the continent. Against this background, the main objective of this study is to answer the following question: Are the disparities in health expenditures among African countries reducing or widening? We selected two proxies for health expenditure to answer this question: the domestic general government health expenditure per capita and the domestic general government health expenditure as a percentage of general government expenditure. The latter was selected as one of the variables of interest based on the 2001 Abuja Declaration. Payne et. al., [12] showed that the main determinants of health expenditure convergence include common policies, among others. Wang [13] earlier revealed that cross-regional policy coordination that promotes resource distribution in the health sector leads to convergence in health expenditure. Hitiris [14] indicated that convergence in countries’ economic performance and living standard leads to convergence in health expenditures. This is because the level of development of countries and their populations’ structure affect their spending on health.

We further contribute to the literature on the field by using the marginal effects of the multinomial logistic regression to identify the factors that explain the probability of belonging to a particular club. In doing so, we construct the quality of governance and the information and communication technology (ICT) variables using principal component analysis (PCA) to examine their impact on the likelihood of being a member of a particular club. We also include trade openness in our models. Trade and governance quality are our main variables of interest, given their functions in determining socioeconomic conditions between and within countries.

The rest of this paper is structured as follows: Sect. " Literature and empirical review" provides the literature and empirical review, Sect. " Methodology" describes the empirical methods used, Sect. " Empirical results and discussion" presents the results, and Sect. " Conclusion and policy recommendations" provides the conclusion and policy recommendations.

## Literature and empirical review

Convergence is a process in which entities move toward uniformity over time [15]. Theoretically, there are two main hypotheses of convergence in the literature. The β-convergence hypothesis suggests that countries with a lower level of health expenditure converge faster than countries with a higher health expenditure and thus catch up with the latter. In contrast, the σ-convergence suggests a decrease in growth dispersion over time [7]. However, the β-convergence test can collapse in the case of stochastic technological progress [16]. In addition, the σ-convergence test can be disproportionately influenced by outliers and short-run shocks [17]. Recently, the stochastic convergence approach has been tested in various fields. In this case, convergence takes place when a particular country’s health expenditure relative to the reference country is stationary, leading to a steady state in the health expenditure level [18]. More recently, the concept of convergence club has gained popularity in the literature. It suggests that convergence occurs between countries with the same structural characteristics and initial conditions. The presence of a convergence club reduces disparities between sub-groups [19].

Previous studies have empirically investigated the convergence hypothesis in health expenditures. For instance, [20] examined the stochastic convergence in a sample of 21 OECD countries for the 1960–1997 period. Using the ADF test and the Kwiatkowski-Phillips-Schmidt-Shin (KPSS) approach, they found no evidence of stochastic convergence of real per capita health expenditure in their selected sample. However, the misspecification errors due to the absence of structural breaks may lead to non-stochastic convergence [21]. In this regard, [21] employed the KPSS stationary test and the minimum LM type statistic to investigate the stochastic convergence with and without breaks and the β-convergence with structural breaks in health expenditure as a share of GDP in 11 OECD countries between 1960 and 2006. The empirical results showed a stochastic convergence pattern across the sample, while the β-convergence occurred in four countries only before the structural break. Odhiambo et. al., [7] considered a linear dynamic panel model to analyze the convergence of health expenditure in Sub-Saharan Africa (SSA) from 2000 to 2011. The authors estimated the model using the GMM-IV method on 41 SSA countries. Their results indicated the presence of absolute and conditional convergence. Their findings also revealed that per capita income, donor funding for health care, and benefiting HIPC debt relief are the main factors that affect the direction and rate of convergence of health expenditures in SSA.

However, other studies have shown that traditional convergence tests possess some flaws. For instance, the ADF is biased and inconsistent under transitional heterogeneity due to omitted variables and endogeneity issues. In addition, health expenditures may follow a non-linear path because it follows the dynamic of income path, which in turn is non-linear [22]. In this regard, [10, 11] have introduced the log-t regression test, which helps test the convergence hypothesis based on a non-linear time-varying factor model. Following [10, 19] analyzed the convergence in health expenditure among 19 OECD countries from 1972 to 2006. Their results showed that health expenditure per capita for the sample diverged at a rate of 0.5 percent. The authors also identified the existence of two convergence clubs but did not find evidence that convergence in health expenditure per capita led to the convergence in health outcomes. Similarly, [6] also used the [10, 11] method to test the convergence hypothesis in public health expenditure in 44 SSA countries between 2000 and 2016. The results show no evidence of convergence for the entire sample. However, the study identified the presence of three convergence clubs.

Therefore, this study uses the methodology developed by [10, 11] on two health expenditure indicators for 40 African countries. The approach is suitable for the study because it incorporates the possibility of individual and transitional heterogeneity or even transitional divergence. This is very important given that the test for convergence using standard panel stationarity tests is not valid [10]. The approach does not also rely on assumptions regarding trend stationarity or stochastic non-stationarity.

## Methodology

We use the methodology developed by [10, 11] to test the convergence of health expenditures in 40 African countries. The PS approach is based on modifying the conventional panel data decomposition of the variables of interest. The panel data ${X}_{it}$ (i = 1,…,N and t = 1,…,T) are decomposed into two components, as follows:

1 $${X}_{it}={\delta }_{it}+ {\rho }_{it}$$

where N is the 40 African countries while T is the period. ${X}_{it}$ is the natural logarithm of the variables of interest, ${\delta }_{it}$ is the systematic component, and ${\rho }_{it}$ is the transitory component. PS further separated common from idiosyncratic elements in the panel by transforming Eq. 1 as follows:

2 $$\begin{array}{cc}X_{it}=\left(\frac{\delta_{it}+\rho_{it}}{\mu_t}\right)\mu_t={\mathrm b}_{it}\mu_t,&\mathrm{for}\;\mathrm{all}\;i,t\end{array}$$

where health expenditure ${X}_{it}$ is decomposed into two time-varying components: the common component ${\mu }_{t}$ and the time-varying systematic idiosyncratic element ${\mathrm{b}}_{it}$. The idiosyncratic component measures the distance between ${X}_{it}$ and the common element and absorbs the error term and the unit-specific component. Furthermore, PS assumes that ${\mu }_{t}$ can follow a trend-stationary process or a non-stationary stochastic trend that dominates the transitory component ${\rho }_{it}$ in the long run. The estimation of ${\mathrm{b}}_{it}$ is central to the approach developed by PS. This is because it delivers information about the transition behavior of particular panel units. PS propose testing of convergence by examining whether ${\mathrm{b}}_{it}$ converges toward $\mathrm{b}$. This can be done by defining the relative transition component as follows:

3 $${h}_{it}= \frac{{X}_{it}}{\frac{1}{N}\sum_{i=1}^{N}{X}_{it}}= \frac{{ \mathrm{b}}_{it}}{\frac{1}{N}\sum_{i=1}^{N}{ \mathrm{b}}_{it}}$$

where ${h}_{it}$ is the transaction path that measures the relative departure of country $i$ from the common steady-state growth path ${\mu }_{t}$. Whenever panel units converge, the relative transaction path ${h}_{it}$ converges to unity such as ${h}_{it} \to 1$ for all i = 1,…,N as $t\to \infty$. At the same time, the cross-sectional variation ${H}_{it}$ of the relative transition path converges to zero as follows:

4 $$H_t=\frac1N\sum_{i=1}^N(h_{it}-{1)}^2\rightarrow0,\;as\;t\rightarrow\infty$$

The cross-sectional mean of the relative transition path ${h}_{it}$ is unity. To construct a formal statistical test for convergence, PS suggest the following semi-parametric model for ${\mathrm{b}}_{it}$

5 $${\mathrm{b}}_{it}= {\mathrm{b}}_{i}+ \frac{{\theta }_{i} {\tau }_{it}}{{L\left(t\right)t}^{a}}$$

where ${\mathrm{b}}_{it}$ is fixed, ${\tau }_{it}$ are $iid$ N (0, 1) across $i$, ${\theta }_{i}$ are idiosyncratic scale elements, $L\left(t\right)$ is a slowly varying function, such as $\mathrm{log}(t)$, so that $L\left(t\right)\to$∞ as $t\to \infty$. The coefficient $a$ captures the speed of convergence. This formulation ensures that ${\mathrm{b}}_{it}$ converges to ${\mathrm{b}}_{i}$ for all $a \ge 0$. The null hypothesis is ${H}_{0}: { \mathrm{b}}_{i} =\mathrm{ b}$ and $a \ge 0$, thereby indicating convergence of the whole sample, while the alternative is ${H}_{1}: { \mathrm{b}}_{i}\ne \mathrm{b}$ for all i, or $a<0,$ suggesting overall divergence. Under convergence, the transition distance ${H}_{t}$ is represented as:

6 $$\begin{array}{cc}H_t\sim\frac A{{L(t)}^2t^{2a}}&\mathrm{as}\;t\rightarrow\infty\end{array}$$

where $A$ is a positive constant. To test the presence of convergence for the sample of countries, PS use the following log $t$ specification:

7 $$\begin{array}{cc}\mathrm{log}\left(\frac{{H}_{1}}{{H}_{t}}\right)-2logL\left(t\right)=\widehat{k}+ \widehat{b}logt+{\mu }_{t},& t= \left[rT\right],\dots , T\end{array}$$

where $L(t)$ is $logt$, ${H}_{t}$ is the cross-sectional variation, and $\frac{{H}_{1}}{{H}_{t}}$ represents the ratio of the cross-sectional variation at the beginning of the sample ${H}_{1}$ (i.e. ${H}_{t}$ at t = 1) divided by the respective variation for every point in time t. -$2log\left(log t\right)$ represents the penalization function that aims at improving the performance of the test under the alternative, and $r>0$. PS suggested setting $r$ є [0.2, 0.3] for a small sample size (T < 50). This is because the extensive Monte Carlo simulations show that this choice of $r$ is stationary in terms of both the size and power properties of the test. The null hypothesis is tested using a one-sided t-test. The latter is robust to heteroscedasticity and autocorrelation of the inequality $a\ge 0$ (using $\widehat{b}= 2a$). The null hypothesis is supported if ${t}_{b}> -1.65$ or if $b$ is either positive or equal to zero. If the $log t$-test is rejected, PS recommend repeating the test procedure according to a clustering mechanism.

### The clustering club merging algorithm

PS propose an empirical algorithm that can be used to identify subgroups of countries that converge to different steady states. This can be described in four steps:

1. Order countries in the sample according to the final values of their health expenditure.
2. Identify a core group of $k$ countries with the highest health expenditure to form the subgroup ${G}_{k}$ for some $N>K\ge 2$. Then run the convergence test. The core group size $k$ is chosen by maximizing ${t}_{b}$ over $k$ following the minimum criteria $\left\{{t}_{b}(k)\right\}>-1.65$.
3. Add each of the remaining countries separately to the core group and run the convergence test for each addition. If ${t}_{b}> -1.65$, then the country is included in the core group. If ${t}_{b}< -1.65$, then the forming of the subgroup is finished and the procedure is repeated to form the next group.
4. Repeat steps 1 to 3 for the remaining countries.

Phillips et. al., [10, 11] noted that using a sign criterion in step 2 may lead to overestimating the initial number of clubs. To remedy this, [11] proposed the algorithm test for adjacent clubs after the clustering algorithm. In this case, if ${t}_{b}$> -1.65, then the clubs are merged at a five percent significance level.

In the next aspect of this study, we employ the multinomial logit model on all the health expenditure variables to investigate the determinants of the probability of belonging to a particular club. Let ${Q}_{i}$ be the random variable that reveals whether $ith$ country is a member of a specific convergence club and $Prob ({Q}_{i}=g)$ is the probability that $ith$ country is a member of the $gth$ convergence group. Additionally, let ${W}_{i}$ be the $Kx1$ conditioning vector of the variables which drive the $ith$ country to belong to a particular club. Furthermore, let ${C}_{g}$ be the $Kx1$ parameter vector where $g$ takes on the values $\{1, 2, 3,\dots , A\}$, with $A$ representing the number of final convergence clubs. The probability that $ith$ country will belong to the $gth$ convergence club is presented as:

8 $$Prob \left({Q}_{i}=g\right)= \frac{exp{(B}_{i}^{l}{C}_{g})}{\sum_{K=1}^{F}{(B}_{i}^{l}{C}_{K})}$$

For identification, coefficient restrictions need to be put based on the final club $p$ (the reference convergence club) as ${C}_{p}$=0. We can calculate the log odds ratio as:

9 $$log\left(\frac{Prob ({Q}_{i}=g)}{Prob ({Q}_{i}=p)}\right)= {B}_{i}^{l} \left({C}_{g}-{C}_{p}\right)={B}_{i}^{l}{C}_{g}$$

where ${C}_{g}$ are the parameter estimates, which are, relative values to the reference club. For each health expenditure measure, we use a different final club as a reference club and estimate the following multinomial logit model for health expenditures:

10 $$log\left(\frac{Prob ({Q}_{i}=g)}{Prob ({Q}_{i}=p)}\right)={C}_{0,g}+{C}_{1,g}\sum_{i=g}^{n=16}{X}_{i}$$

${X}_{i}$ represents a vector of the determinants of the dependent variables used.

### Data and variables description

The data was extracted from the World Bank Development Indicator (WDI) and the World Governance Indicator (WGI), all provided by the World Bank. The variables were selected based on previous literature and the availability of data, for the period 2000–2019. We consider two proxies for health expenditure: domestic general government health expenditure per capita and domestic general government health expenditure as a percentage of general government expenditure. All the variables with nominal values were deflated using the GDP deflator collected from the World Bank. We use the GDP deflator because it covers the overall economy and is a more reliable measure of the overall inflation rate in the country [23].

We did not have an issue related to missing observations. We multiplied the external health expenditure per capita (PPP) by the total population. We further divided external health expenditure with the gross domestic product to obtain external health expenditure as a percentage of GDP. Details about all the variables used in the study are reported in Panel A of Appendix, while the list of countries is in Panel B of Table 6 Appendix.

## Empirical results and discussion

### Empirical results

#### Correlation matrix and principal component results analysis

The results presented in Panel A of Table 1 reveal that the correlations among the governance indicators are statistically significant. Due to the high degree of collinearity observed between them, we construct the governance quality variable using PCA. The results are presented in Table 1 Panel A. We retain the principal component with an eigenvalue greater than one and the eigenvector associated with variables whose loading exceeded 0.40 in absolute value [24], which in this study is component one. Similarly, the correlation between the information and communication technology proxies reported in Panel B of Table 1 is also high and significant. Component 1 is selected because its eigenvalue is greater than one.

**Table 1:** Correlation matrix and principal component analysis results

**Panel A: Governance indicators correlation matrix and principal component analysis results**							
	GE	PSAVT	CC	RQ	RL	VA	
GE	1.000						
PSAVT	0.639 0.000	1.000					
CC	0.847 0.000	0.677 0.000	1.000				
RQ	0.903 0.000	0.672 0.000	0.823 0.000	1.000			
RL	0.904 0.000	0.754 0.000	0.886 0.000	0.885 0.000	1.000		
VA	0.682 0.000	0.598 0.000	0.709 0.000	0.727 0.000	0.783 0.000	1.000	
**Principal component results**							
**Components**	**Eigenvalue**	**Difference**	**Proportion**	**Cumulative**			
Component 1	4.834	4.380	0.806	0.806			
Component 2	0.455	0.085	0.076	0.882			
Component 3	0.370	0.188	0.062	0.943			
Component 4	0.181	0.086	0.030	0.973			
Component 5	0.095	0.030	0.016	0.989			
Component 6	0.065		0.011	1.000			
**Principal component eigenvector results**							
**Variables**	**Component 1**	**Component 2**	**Component 3**	**Component 4**	**Component 5**	**Component 6**	**Unexplained**
GE	0.423	-0.287	-0.321	0.244	-0.575	0.495	0
PVST	0.359	0.897	-0.125	0.148	0.080	0.151	0
CC	0.419	-0.100	-0.175	-0.841	0.231	0.153	0
RQ	0.422	-0.318	-0.149	0.459	0.696	-0.060	0
RL	0.442	-0.013	-0.059	-0.012	-0.350	-0.824	0
VA	0.378	-0.050	0.908	0.020	-0.056	0.163	0
**Panel B: ITC indicators correlation matrix and principal component analysis results**							
	NET	CEL					
NET	1.000						
CEL	0.805 0.000	1.000					
**Principal Component results**							
**Components**	**Eigenvalue**	**Difference**	**Proportion**	**Cumulative**			
Component 1	1.805	1.609	0.902	0.902			
Component 2	0.195		0.0977	1.0000			
**Principal component eigenvector results**							
**Variables**	**Component 1**	**Component 2**	**Unexplained**				
NET	0.7071	0.7071	0				
CEL	0.7071	-0.7071	0				

Source: Author’s computation

#### Descriptive statistics

The descriptive statistics are presented in Table 2. On average domestic general government health expenditure accounted for 7.079 percent of the general government expenditure, ranging from a minimum value of 0.633 to a maximum of 18.287 percent. These results suggest differences in the countries’ political commitment to the health sector. The average per capita domestic general government health expenditure stood at about US$ 100.155, with variations across countries, as shown by the deviation from the mean of US$ 148.602.

**Table 2:** Descriptive statistics of variables of the full sample

Variables	Mean	Std. Dev	Min	Max
**Dependent variables**				
Domestic general government health exp. (GGHE/GE)	7.079	3.490	0.633	18.287
Domestic general government health expenditure per capita (DGHEp)	100.155	148.602	0.277	881.148
**Independent variables**				
Real GDP per capita (RGDPp)	5316.538	5767.766	715.454	41,249.490
Population above 65 year old (POP ≥ G65)	3.378	1.398	1.871	11.999
Population below 15 years old (POP˂15)	41.267	6.488	17.260	50.264
Urban population (URB)	42.218	16.900	8.246	89.741
External health expenditure (%of GDP) (EXTHE)	.208508	0.016	0.000	0.537
Trade (TRD)	66.361	28.426	1.219	175.798
Life expectancy at birth (LE)	58.852	7.709	39.441	76.880
Infant mortality rate (IMR)	58.376	24.767	12.500	138.100
People using at least basic sanitation service (SAN)	36.508	23.792	4.192	96.377
Incidence of HIV (HIV)	2.212	3.490	0.010	21.680
Incidence of tuberculosis (TB)	281.710	274.368	11.000	1590.000
Governance quality (INS)	4.493	2.199	0.000	10.235
Information and communication technology (ICT)	1.341	1.343	0.000	5.939

Source: Authors’ computation from WDI, WGI and ADI (World Bank)

On average, GDP per capita was about US$5316.538, with a standard deviation of about US$5767.766 per capita, which reveals disparities in income distribution across the selected sample. The average external health expenditure was about 0.208 percent of GDP. The average TB incidence was about 281.710 per 100,000 people, while on average, HIV incidence was about 2.212 per 1,000 uninfected population. Average life expectancy was about 59 years, ranging from 37 to 77 years. The average infant mortality rate was about 58 per 1,000 live births. On average, about 41 percent of the population was below 15 years old, while only about 3.378 percent was 65 years and above.

Moreover, about 42 percent of the population lived in urban areas, indicating a rapid urbanization in the continent. About 37 percent of the population used basic sanitation services on average, with a maximum of about 96 percent. The average trade was about 66 percent of GDP, with a minimum of 1.219 and a maximum of 175.798 percent. The mean of governance quality is 4.493, while the mean of ICT is 1.341, suggesting low connectivity across the continent.

#### Convergence test results

The convergence results are presented in Table 3. The null hypothesis of full panel convergence is rejected at 5 percent significant level for the two variables of interest, given their t-statistics of -24.683 and -12.553 respectively are below the critical value of -1.65. We further applied the club clustering test. The results show the existence of two initial convergence clubs and two divergent countries regarding the domestic general government health expenditure as a percentage of general government expenditure variable. We also found the presence of three convergence clubs for the domestic general government health per capita variable. We further perform the club merging test. In terms of the domestic general government health expenditure as a percentage of general government expenditure variable, we found that the initial clubs cannot be merged to form larger convergence clubs. Therefore, the initial two convergence clubs are the final clubs of 27 and 11 countries and two divergent countries. In the case of the domestic general health expenditure per capita, the merging test results show the reduction of the initial clubs into two final clubs of 25 and 15 countries.

**Table 3:** Convergence and final club classification results

Sample	Countries	b ^ *Coeff*	SE	t-stat
**Panel A: Domestic general government health exp. (GGHE/GE)**				
Overall (40)	All the selected countries in the sample	-1.088^a^	0.044	-24.683
Cub 1 (21)	Algeria | Angola | Botswana | Burkina Faso | Burundi | Cabo Verde | Congo, Dem. Rep. | Cote d’Ivoire | Equatorial Guinea | Gabon | Ghana | Guinea | Kenya | Madagascar | Mauritania | Mauritius | Morocco | Namibia | Niger | Rwanda | South Africa | Sudan | Eswatini | Tanzania | Togo | Tunisia | Zambia	0.118	0.075	1.568
Club 2 (11)	Benin | Central African Republic | Chad | Comoros | Congo, Rep. | Gambia, The | Mali | Nigeria | Senegal | Sierra Leone | Uganda	0.918	0.111	8.307
Club 0 (not converging group) (2)	Cameroon | Guinea-Bissau	-4.156^a^	0.468	-8.882
**Club Merging**				
Club 1 + 2		-0.503^a^	0.026	-19.716
**Final club classifications**				
Final club 1 (21)	Algeria | Angola | Botswana | Burkina Faso | Burundi | Cabo Verde | Congo, Dem. Rep. | Cote d’Ivoire | Equatorial Guinea | Gabon | Ghana | Guinea | Kenya | Madagascar | Mauritania | Mauritius | Morocco | Namibia | Niger | Rwanda | South Africa | Sudan | Eswatini | Tanzania | Togo | Tunisia | Zambia	0.118	0.075	1.568
Final club 2 (11)	Benin | Central African Republic | Chad | Comoros | Congo, Rep. | Gambia, The | Mali | Nigeria | Senegal | Sierra Leone | Uganda	0.918	0.111	8.307
Club 0 (not converging group) (2)	Cameroon | Guinea-Bissau	-4.156^a^	0.468	-8.882
**Panel B: Domestic general government health expenditure per capita (DGHEp)**				
Overall (43)	All the selected countries in the sample	-0.312^a^	0.025	-12.553
Cub 1 (12)	Algeria | Burkina Faso | Congo, Dem. Rep. | Congo, Rep. | Cote d’Ivoire | Equatorial Guinea | Gambia, The | Guinea | Mauritania | Mauritius | South Africa | Togo	2.716	2.288	1.187
Club 2 (13)	Botswana | Burundi | Cabo Verde | Gabon | Ghana | Kenya | Morocco | Namibia | Niger | Rwanda | Eswatini | Tunisia | Zambia	-0.032	0.033	-0.962
Club 3 (15)	Angola | Benin | Cameroon | Central African Republic | Chad |Comoros | Guinea-Bissau | Madagascar | Mali | Nigeria | Senegal | Sierra Leone | Sudan | Tanzania | Uganda	3.283	2.208	1.487
**Club Merging**				
Club 1 + 2		1.562	0.024	65.671
Club 2 + 3		-2.556^a^	0.058	-44.472
**Final club classifications**				
Final club 1 (25)	Algeria | Botswana | Burkina Faso | Burundi | Cabo Verde | Congo, Dem. Rep. | Congo, Rep. | Cote d’Ivoire | Equatorial Guinea | Gabon | Gambia, The | Ghana | Guinea | Kenya | Mauritania | Mauritius | Morocco | Namibia | Niger | Rwanda | South Africa | Eswatini | Togo | Tunisia | Zambia	1.562	0.024	65.671
Final club 2 (2)	Angola | Benin | Cameroon | Central African Republic | Chad |Comoros | Guinea-Bissau | Madagascar | Mali | Nigeria | Senegal | Sierra Leone | Sudan | Tanzania | Uganda	3.283	2.208	1.487

^a^indicates rejection of the null hypothesis of convergence and club convergence merging. *SE* Standard error

Source: Authors’ computation using WDI data

Additionally, the results reveal that the final club 2 of the general government health expenditure per capita model is mainly composed of countries affiliated to the final club 2 and the divergent group in the general government health expenditure as a percentage of the general government expenditure model. These results are consistent with [6], who also found the absence of overall convergence in Sub-Saharan Africa (SSA). However, the results contradict [7], who found evidence of absolute and conditional convergence for public health expenditure as a percent of government expenditure in SSA countries.

In addition, we measured the gaps between the final clubs for both variables of interest. This allows us to understand the disparities between the different clubs better. The results are reported in Table 4. The means values suggest that countries in the final club 1 spent on average 7.786 of their general government expenditure on health. However, countries in the final club 2 and the divergent group spent an average of 5.583 and 5.757 percent of their government expenditure on health, respectively. These findings show gaps between the clubs, especially between countries in clubs 1 and the rest. The gap between club 2 and the divergent group is small. The results also suggest that there are disparities within the clubs.

**Table 4:** Average of health expenditure measures by final clubs, 2000–2019

	**Domestic general government health exp. (% of GE)**			**Domestic general government health expenditure per capita**		
**Clubs**	**Mean** **(Sd. Dev.)**	**Min**	**Max**	**Mean** **(Sd. Dev.)**	**Min**	**Max**
Final club 1	7.786 (3.549)	1.334	18.287	1.910 (1.276)	0.062	5.353
Final club 2	5.583 (2.496)	1.344	15.031	1.159 (0.580)	0.121	3.223
Club 0 (not converging group)	5.757 (4.381)	0.633	15.140			
Total	7.079 (3.490)	0.633	18.287	1.628 (1.129)	0.062	5.353

Source: Authors’ own computation

**Fig. 1:**
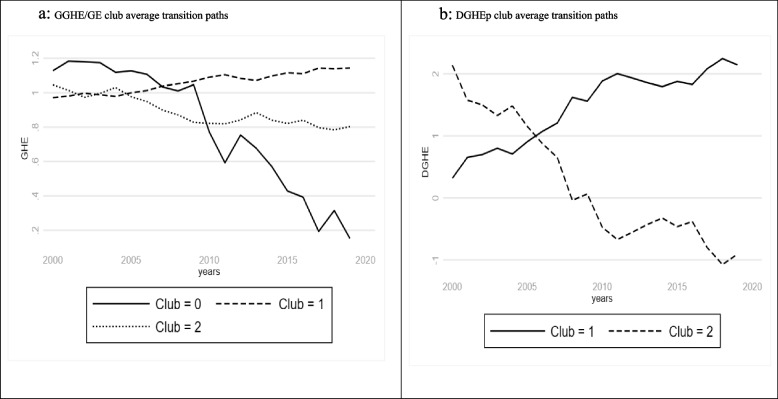
Average transition paths for the clubs- 2000–2019. **a** GGHE/GE club average transition paths. **b** DGHEp club average transition paths

Furthermore, in terms of the domestic general government expenditure per capita, there is also a gap between the two clubs. As shown in Table 3, countries belonging to the clubs with lower health spending are similar in both models. World Health Organization Regional Office for Africa [2] showed that most countries belonging to the final club 2 and the divergent group are characterized by challenging macroeconomic context and low levels of GDP, which limit their capacities to mobilize domestic financial resources for their health sectors. Moreover, most of these countries face other critical challenges, including internal or post-conflict, poverty, corruption, bad governance, which significantly limit public funds for health. Figure 1 shows the differences between the averages transitional behavior of the clubs for the variables of interest. In Fig. 1a, on average, we observed a shape decline in GGHE/GE for club 0 (which is composed of the diverging countries: Cameroon and Guinea Bissau) from the time when the global financial crisis took hold. However, on average, GGHE/GE in Club 1 increases over time, while it fluctuates in club 2. Figure 1b, which displays the average transitional behavior of the two converging clubs, reveals that on average DGHEp decreases in Club 2 over time, while it increases in Club 1. For both clubs, the decline starts to become shape in 2005.

#### Marginal effect results

Table 5 reports the results of the marginal effects for the two health expenditure models. Contrary to the multinomial logistic model results, the marginal effects determine the direction and magnitude of the relationship between the dependent variable and the explanatory one. They measure the probability of one country belonging to a particular club when a specific explanatory variable changes by one unit, while holding other variables constant [25, 26]. The two models are statistically significant, with *P*-values < 0.000. In this case, we reject the hypothesis that the coefficients of the overall variables for the different equations are jointly equal to zero. The marginal effects of the variables reported show mixed results.

**Table 5:** Marginal effects results

Variables	Clubs	GGHE/GE	DGHEp
lRGDPp	Final club 1	0.209*** (0.035)	-0.093*** (0.030)
	Final club 2	-0.082** (0.033)	0.093*** (0.030)
	Divergent gr	-0.128*** (0.023)	
lPOP<15	Final club 1	-0.759** (0.259)	-1.413*** (0.295)
	Final club 2	1.229*** (0.294)	1.413*** (0.295)
	Divergent gr	-0.471** (0.182)	
lPOP ≥ 65	Final club 1	0.467*** (0.121)	0.038 (0.116)
	Final club 2	-0.091 (0.128)	-0.038 (0.116)
	Divergent gr	-0.377*** (0.089)	
lURB	Final club 1	-0.807*** (0.075)	-0.259*** (0.052)
	Final club 2	0.196** (0.071)	0.259*** (0.052)
	Divergent gr	0.611*** (0.108)	
lEXHE	Final club 1	-0.420 (0.316)	-0.177 (0.228)
	Final club 2	0.544 (0.369)	0.177 (0.228)
	Divergent gr	-0.124 (0.173)	
lTRD	Final club 1	0.167*** (0.030)	0.416*** (0.040)
	Final club 2	-0.035 (0.028)	-0.416*** (0.040)
	Divergent gr	-0.132*** (0.019)	
lSAN	Final club 1	0.003 (0.025)	0.139*** (0.026)
	Final club 2	0.035 (0.027)	-0.139*** (0.026)
	Divergent gr	-0.038** (0.015)	
lTB	Final club 1	0.121*** (0.028)	-0.017 (0.023)
	Final club 2	-0.131*** (0.030)	0.017 (0.023)
	Divergent gr	0.010 (0.016)	
HIV	Final club 1	0.055*** (0.011)	0.051*** (0.012)
	Final club 2	-0.088*** (0.013)	-0.051*** (0.012)
	Divergent gr	0.032*** (0.005)	
lIMR	Final club 1	0.253** (0.103)	-0.192* (0.101)
	Final club 2	-0.680*** (0.123)	0.192* (0.101)
	Divergent gr	0.427*** (0.081)	
lLE	Final club 1	2.383*** (0.318)	0.578* (0.341)
	Final club 2	-3.381*** (0.323)	-0.578* (0.341)
	Divergent gr	0.999*** (0.195)	
llCT	Final club 1	-0.018 (0.014)	-0.037** (0.014)
	Final club 2	0.016 (0.013)	0.037** (0.014)
	Divergent gr	0.002 (0.005)	
lINS	Final club 1	-0.087** (0.033)	-0.045 (0.034)
	Final club 2	0.044 (0.035)	0.045 (0.034)
	Divergent gr	0.043** (0.014)	
**No. of observation**		**785**	**762**
***P*-Values**		**0.0000**	**0.0000**

Source: Authors’ own computations

Note: ***, **, and * represent statistical significance at 1%, 5%, and 10%

#### Domestic general government health expenditure as a percentage of general government expenditure

The overall pattern for most control variables included in the GGHE/GGE model is as expected. For example, a unit increase in the initial log RGDPp increases the probability of belonging to club 1 by 0.21 log points. However, it reduces the likelihood of being affiliated with the final club 2 and the divergent club by 0.08 and 0.13 log points, respectively. Surprisingly, a similar pattern is observed for variables such as the incidence of HIV, lTB, and lIMR. The findings can be explained by the high incidence of HIV and TB in most countries included in the final cub 1 and the divergent group [1]. However, when the initial lPOP˂15 increases by one percent, there is a lower probability of belonging to the final club 1 and the divergent group. However, there is a higher probability of belonging to final club 2. The marginal effects for the other control variables also show mixed results.

The results also reveal that a unit increase in trade increases the probability of belonging to the final club 1 by about 0.17 log points. But it lowers the likelihood of membership to the divergent and final clubs 2. Africa Regional Integration Index [27] report shows that countries such as Eswatini, Namibia, and South Africa (all belonging to the final club 1) were the best performer on trade integration in Africa between 2000 and 2019. Many countries in the final club 1 were also average performers on trade integration in the continent and they had average shares of intra-regional trade. However, all countries in the divergent group had low levels of trade integration in Africa. The marginal effects results of ICT are insignificant for all the clubs. Surprisingly, a unit improvement in the quality of governance reduces the probability of belonging to the final club 1 by 0.09 log points. But it increases the likelihood of membership to the divergent group by 0.04 log points. It is important to note that the overall marginal effect for the final club 1 might be overshadowed by a tremendous gap between the countries within the club. For instance, countries such as Mauritius, Ghana, and South Africa had the highest scores on the rule of law in 2017, while Botswana and Cabo Verde scored relatively well on transparency and accountability. However, countries such as Sudan, DRC, and Madagascar scored substantially worse in the continent [28].

#### Domestic general government health expenditure per capita

Similarly, the marginal effects of most of the control variables included in the DGGHEp model are as expected. For instance, a unit increase in the initial lPOP˂15 reduces the likelihood of belonging to club 1 by 1.41 log points. Still it increases the probability of membership to the final club 2 by 1.41 log points. A similar pattern is recorded in terms of urbanization and infant mortality rate. Moreover, a unit increase in lSAN, lLe, and HIV incidence leads to a higher probability of affiliation to final club 1, but a lower one in terms of membership to final club 2. It is crucial to note that countries such as South Africa, Botswana, and Namibia (all belonging to the final club 1) have a high incidence of HIV/AIDS [1]. This may explain the positive affiliation with the final club 1 regarding the HIV incidence variable. The effects of the rest of the control variables show mixed results.

When analyzing our variable of interest, the results show that a unit increase in trade increases the likelihood of belonging to the final club 1 by about 0.42 log points. However, it reduces the probability of membership to the final club 2 by about 0.42 log points. As mentioned above, some countries in the final club 1 were classified as the best performer in trade integration in Africa. These include Eswatini, Namibia, and South Africa. Other countries such as Gabon, Côte d’Ivoire, Botswana, the Gambia, Ghana, and Congo were average performers in trade integration. Many countries in the final club 1 scored within the intra-regional trade average.

Regarding the final club 2, most countries were classified as low performers, with lower shares of intra-regional trade [27]. There is a lower probability of about 0.04 log points of belonging to the final club 1, when lICT increases by one unit. But the likelihood of belonging to the final club 2 is higher when lICT increases by the same unit. These results can be explained by the gap in ICT between countries belonging to final club 1 [1]. The results of institutional quality are insignificant for all the final clubs.

## Discussion

The study’s findings suggest that Africa’s health expenditures display a divergence. The absence of overall convergence comes from the existence of country-specific factors that cause disparities in health spending, even among countries belonging to the same regional economic blocs. The results also show convergence among sub-groups for both variables of interest. The results further show that real GDP per capita as a proxy of income, young population, aging population, urbanization, TB and HIV incidence are the determinants of convergence in health expenditures [7].

The findings also imply that trade positively affects the likelihood of converging to the final club with the highest average domestic general government health expenditures. These results are consistent with [4], who showed that increased trade openness provides fiscal space for the health sector. Therefore, the results suggest that the health sectors in African countries could gain from opening up for trade at the regional, continental and international levels. Convergence in domestic health expenditures could be enhanced due to improved trade openness. Africa is among the worst governance performers [28]. Several countries face massive corruption, internal or post-conflicts, weak rule of law, and civil unrest.

The negative effects of governance quality for the final club 1 for the domestic general government health expenditure as a percentage of general government expenditure model suggests existence of the tremendous gap between countries belonging to that club. Nevertheless, our findings show that governance quality explains the club formation in terms of the domestic general government health expenditure as a percentage of general government expenditure. Policy efforts towards improving trade openness and the quality of governance will be a step towards uniformity in health expenditures among the final clubs.

## Conclusion and policy recommendations

This study investigated the convergence hypothesis for two health expenditure variables among 40 African countries spanning 2000–2019. We employed the non-linear time-varying factor model. Our results show an absence of full sample convergence for the two health expenditure indicators used. This means that despite the efforts made at all levels, disparities in health expenditures remain a concern in Africa. Two convergence clubs were identified within each model considered. The graphical representation of the average values shows gaps between the clubs. The disparities among clubs were small in terms of domestic general government health expenditure per capita.

We then examined the factors that affect club formation by computing and reporting the marginal effects of the multinomial logic model. In doing so, we first create the governance quality variable using PCA. Our results show mixed results for the different clubs. However, as expected, the young population and life expectancy at birth considerably impact the probability of belonging to a particular club for all the dependent variables. The results also show that trade openness positively affects the likelihood of converging to clubs with the highest average domestic general government health expenditures. But it decreases the probability of converging to the clubs with lower levels of domestic general government health expenditures. However, a different pattern is observed regarding the quality of governance variable. The latter variable’s effects are insignificant regarding the general government health expenditure per capita.

This study recommends that the efforts to reduce health expenditure disparities among African countries should vary across clusters and not be generalized. Efforts to improve health expenditure convergence in Africa should also encourage trade openness at regional, continental and international levels. In addition, policymakers should pursue avenues for improving governance quality among countries.

## Data Availability

The datasets used in the current study are publicly available and can be found in the World Bank Databank website.

## Appendix

**Table 6:** List of variables and countries

Variables	Description		Sources
**Panel A: List of variables and description**			
**Health expenditure variables**			
GGHE/GE	Domestic general government health expenditure (% of general government expenditure)		WDI database
DGHEp	Domestic general government health expenditure per capita, PPP (current international $)		WDI database
**Explanatory variables**			
URB	Urban population (% of total population)		WDI database
POP < 15	Population ages 0–14 (% of total)		WDI database
POP ≥ 65	Population ages 65 and above (% of total)		WDI database
RQ	Regulatory Quality		WGI database
RL	Rule of Law		WGI database
GE	Government Effectiveness		WGI database
PSAVT	Political Stability and Absence of Violence/Terrorism		WGI database
CC	Control of Corruption		WGI database
VA	Voice and Accountability		WGI database
EXHE	External health expenditure per capita, PPP (current international $)		WDI database
RGDPp	GDP per capita, PPP (constant 2017 international $)		WDI database
TRD	Trade (% of GDP)		WDI database
HIV	Incidence of HIV, all (per 1,000 uninfected population)		WDI database
TB	Incidence of tuberculosis (per 100,000 people)		WDI database
IMR	Mortality rate, infant (per 1,000 live births)		WDI database
LE	Life expectancy at birth, total (years)		WDI database
SAN	People using at least basic sanitation service (% of population)		WDI database
NET	Individual using the internet (% of population)		WDI database
CEL	Mobile cellular subscriptions (per 100 people)		WDI database
**Panel B: List of countries**			
Algeria	Guinea	Togo	
Angola	Guinea-Bissau	Tunisia	
Benin	Kenya	Uganda	
Botswana	Madagascar	Zambia	
Burkina Faso	Mali		
Burundi	Mauritania		
Cameroon	Mauritius		
Cabo Verde	Morocco		
Central Afr. Rep	Namibia		
Chad	Niger		
Comoros	Nigeria		
Congo, Dem. Rep	Rwanda		
Congo, Rep	Senegal		
Cote d’Ivoire	Sierra Leone		
Equatorial Guinea	South Africa		
Gabon	Sudan		
The Gambia	Swaziland		
Ghana	Tanzania		

Countries were selected on the basis of data availability

*WDI* World Bank’s World Development Indicators, *WGI* World Bank’s Worldwide Governance Indicators
